# Editorial: Neuroinflammation and neuroimmune response in experimental MCAO and ischemic stroke

**DOI:** 10.3389/fnagi.2023.1195395

**Published:** 2023-05-11

**Authors:** Yingzhe Zhao, Yuge Wang, Deqin Geng, Yanqiang Wang

**Affiliations:** ^1^Department of Neurology II, Affiliated Hospital of Weifang Medical University, Weifang, Shandong, China; ^2^Department of Neurology, The Third Affiliated Hospital of Sun Yat-sen University, Guangzhou, China; ^3^Department of Neurology, Affiliated Hospital of Xuzhou Medical University, Xuzhou, Jiangsu, China

**Keywords:** ischemic stroke, neural inflammation, neuroprotection, blood-brain barrier, inflammation

Stroke, is one of the most significant medical challenges worldwide, because it carries a serious health and economic burden (Karikari et al., 2018). The global disease burden study indicates that the number of cases of new stroke, epidemics, and deaths per year remains high (Feigin et al., 2016). Stroke, especially ischemic stroke, is caused primarily by an interruption in cerebral blood flow, which induces severe neurologic deficits (Qin et al., 2022). Intravenous administration of tissue plasminogen activator (t-PA) and endovascular treatment is currently available for recanalizing the blood flow, but are limited by a narrow therapeutic time window and the potential adverse effects of intracranial hemorrhage and are influenced by technical capabilities and hospital conditions (Mosconi and Paciaroni, 2022). However, evidence of the benefit of these treatment options for ischemic stroke is usually limited. Despite the promising neuroprotective strategies, the mechanism of neuronal death after ischemia is still unclear, and further clinical research is required. Thus, it is paramount to further clarify the mechanisms of ischemic stroke and develop new therapies against the disease.

Increasing evidences show that neuroinflammatory response to acute cerebral ischemia is a major factor in the pathobiology and prognosis of stroke (Shaheryar et al., 2021; Stuckey et al., 2021; Puleo et al., 2022). Inflammatory activity begins after ischemia in the acute phase, followed by excitotoxicty; free-radical production; neuron necrosis; apoptosis; disruption of the blood-brain barrier (BBB) and of neurovascular units; microcirculatory dysfunction; brain edema; hemorrhagic transformation (Jurcau and Simion, 2021); resident microglia activation; infiltration of inflammatory cells such as T cells, monocytes, neutrophils, and different inflammatory cells (Qiu et al., 2021); and discharge of immune mediators including cytokines and chemokines. All of these factors collectively worsen neurological outcomes. in later stages of pose-stroke, these inflammatory mediators support tissue repair and functional recovery. Recent studies have implicated the pathological processes of ferroptosis, mitochondrial metabolism, imbalanced gut microbial community, or dysbiosis in ischemic stroke (Zhou et al., 2021; Zhang et al., 2022). Therefore, inhibiting post-stroke neuroinflammation can alleviate ischemic brain injury and is the basis for future research and novel therapeutics for ischemic stroke.

Clinical research mainly focuses on infiltrating peripheral immune inflammatory cells (Jayaraj et al., 2019). Variations in their counts and ratios is associated with early and late course and prognosis of ischemic stroke and/or treatment with recombinant t-PA and/or thrombectomy (Mosconi and Paciaroni, 2022). These studies have also emphasized that neutrophil-specific gene expression patterns may contribute to poor treatment responses (Bui et al.). High ratio of monocytes to high-density lipoprotein is associated with hemorrhagic transformation in acute ischemic stroke on intravenous thrombolysis (Xia et al.). Matrix metalloproteinase-9 (MMP-9) and brain-derived neurotrophic factor (BDNF) are closely related to the prognosis of ischemic stroke in a time-dependent manner (Li et al.). However, it is worth noting that these findings suggest that the clinical application value of these effects depend on infiltrating into the ischemic site and peak time, location of infarcts, time period since occurrence, severity of ischemia, and stroke subtype. The poor quality and small quantity of included clinical research articles have influenced the assessment. Therefore, constructing a scientific evaluation model to predict stroke by inflammatory factors needs further validation in multi-center, large-sample, and high-quality studies.

The study of neuroinflammation in animal experiments provides a theoretical basis to explore the pathogenesis and progression of ischemic stroke. Additionally, it is beneficial to the clinical transformation of new diagnostic, prognostic, and therapeutic neuroprotective strategies for stroke (Kumar and Aakriti, 2016; Sommer, 2017). Experimental research on animal models mainly focuses on resident cells, the release of inflammatory factors, and signaling pathway (Ma et al., 2020). The studies included in this special edition reviews the inflammatory factors and signal pathways involved in stroke. It was found that VEGF, TGF-β, Ccl19, Ccl24, IL17a, IL3, and complement C5 were the most frequently involved inflammatory factors were. These inflammatory factors aggravate ischemic brain injury and activate corresponding pathways and promote the release of inflammatory mediators (Hammond et al.). The inflammatory signaling pathways involved in ischemic brain injury are complex. AMPK/AKT/GSK3β pathway, SLC7A11/GSH/GPX4 pathway, and PDGFRβ/PI3/Akt pathway are all involved in the regulation of ischemic brain injury injury (Li et al.; Xu et al.). Different signaling pathways can exacerbate neuroinflammation-mediated ischemic brain injury. The MCAO/R (middle cerebral artery occlusion-reperfusion) model is one of the models that most closely simulate ischemic stroke (Mccabe et al., 2018; Wimmer et al., 2018). However, ischemic stroke is a heterogeneous disease with complex pathophysiology, and it is thus impossible to mimick all aspects of human stroke in a single animal model, other factor might be the selection of animal stroke models, in young animals without any comorbidity such as hypertension, diabetes, and obesity (Fluri et al., 2015; Sommer, 2017). The rate of successful translation of preclinical stroke research has been very low. It is very important to choose an appropriate stroke model and ensure that the scientific, standardized study design of preclinical trials can increase the translational potential of stroke models (Sommer, 2017; Narayan et al., 2021).

Neuroinflammation is a key player in the progression of ischemic brain injury. However, whether this mechanism exerts beneficial or detrimental effects in ischemic stroke pathology may depend on the time following cerebral ischemia (Jayaraj et al., 2019). Neuroinflammation is a complex phenomenon governed by many factors such as activated astrocytes, microglia and endothelial cells, cytokines, chemokines, and reactive oxygen species (Qin et al., 2019). Neuroinflammation maybe an interesting target for therapeutic intervention. However, the research findings of neuroinflammation in preclinical animal models and clinical transformation are far from expected (Candelario-Jalil and Paul, 2021). Hence, understanding the time-dependent role of neuroinflammation in stroke pathophysiology and performing basic research by using stroke models combined with real-world clinical conditions (Fluri et al., 2015) will certainly provide more data on the development of novel neuroprotective strategies for post-stroke inflammation that could help alleviate the global burden of stroke.

## Author contributions

All authors have contributed to the writing, editing, and approved the final version of the text for submission.
